# OxLDL/LOX-1 mediated sex, age, stiffness, and endothelial dependent alterations in mouse thoracic aortic vascular reactivity

**DOI:** 10.3389/fphys.2024.1471272

**Published:** 2024-11-05

**Authors:** Trevor S. Wendt, Saema Ansar, Rayna J. Gonzales

**Affiliations:** ^1^ Department of Basic Medical Sciences, University of Arizona, Phoenix, AZ, United States; ^2^ Applied Neurovascular Research, Neurosurgery, Department of Clinical Sciences, Lund University, Lund, Sweden

**Keywords:** endothelium, oxidized low-density lipoprotein (OxLDL), lectin-like oxLDL receptor 1 (LOX-1), sex differences, age, vasoreactivity

## Abstract

Elevated plasma levels of oxidized low-density lipoprotein (oxLDL) are a risk factor and key component that accelerates and worsens cardiovascular disease fueling inflammation, plaque buildup and vascular damage. OxLDL can elicit its detrimental action via lectin-like oxLDL receptor 1 (LOX-1). In this study, we determined whether oxLDL, via LOX-1, alters aortic vascular reactivity and determined if sex and age differences exist. Thoracic aortic endothelium-intact or -denuded ring segments were isolated from 7 to 12 months old intact C57BL/6J female and male mice and pre-incubated with oxLDL *ex vivo* (50ug/dL; 2 h). Using wire myography, cumulative concentration-response curves to phenylephrine (PE) were generated to determine contractile responses. From these curves, the EC50 was determined and used to contract rings to assess acetylcholine (ACh) dependent relaxation. Calculated aortic stiffness and remodeling were also assessed. BI-0115 (10 μ M; selective LOX-1 inhibitor) was used to determine LOX-1 dependence. We observed differential sex, age, endothelial cell, and LOX-1 dependent alterations to the efficacy of PE-induced contractile responses and ACh-mediated vasorelaxation in thoracic aortic rings following oxLDL exposure. Additionally, we observed a distinct sex and age effect on thoracic aortic stiffness following exposure to oxLDL. There was also a sex effect on calculated vessel diameter, as well as an age effect on oxLDL-mediated aortic remodeling that was LOX-1 dependent. Thus, LOX-1 inhibition and the resulting attenuation of oxLDL/endothelial-mediated alterations in aortic function suggests that there are differential sex differences in the role of oxLDL/LOX-1 in the thoracic aorta of middle-aged male and female mice. NEW and NOTEWORTHY. We investigated the effects of oxLDL via the LOX-1 receptor on murine thoracic aortic vasoreactivity, stiffness, and remodeling across age and sex. Acute exposure to oxLDL led to altered vasoreactivity, endothelial dysfunction, and changes in aortic stiffness and remodeling. These effects were in-part age, sex, endothelial, and LOX-1 dependent. This study reveals potential complex interactions in oxLDL/LOX-1-mediated vascular responses that could serve as potential therapeutic intervention for vascular diseases such as atherosclerosis and stroke.

## 1 Introduction

Elevated oxidized low-density lipoprotein (oxLDL) is a risk factor and key factor in the development of vascular pathogenesis associated with atherosclerosis and stroke (Trpkovic et al., 2015; Xu et al., 2022). The rising prevalence of dyslipidemia, which affects not only aged populations (Liu et al., 2023) but also younger cohorts due to increased prevalence of poor diet and lack of exercise, has sparked interest in better understanding the molecular and functional effects of elevated oxLDL plasma levels on the vascular wall. One of the preceding indications of development of oxLDL-induced pathology is augmented endothelial dysfunction which can ultimately predispose vessels to structural wall changes (Gimbrone and García-Cardeña, 2016; Ross, 1993). A defining feature of oxLDL-mediated processes, closely linked to endothelial dysfunction, is oxidative stress (Valente et al., 2014). Mechanisms associated with the complex regulation of redox balance by oxLDL has emerged as a pivotal focus in the study of cardiovascular diseases. This is further convoluted by the addition of inflammation which has also been investigated in the context of atherosclerosis, aneurysmal formation, and stroke, and demonstrates the intricate mechanisms through which oxLDL may impact vascular function and health.

OxLDL is a byproduct of lipid peroxidation and exerts its effects on a number of cell types including endothelial cells, platelets, macrophages, fibroblasts, and smooth muscle cells through lectin-like oxLDL receptor 1 (LOX-1) (Pirillo et al., 2013; Truthe et al., 2024). LOX-1 is described as a type II integral membrane glycoprotein receptor that was initially cloned from bovine aortic endothelial cells and human lung by Sawamura et al. (1997). In addition to oxidative stress and inflammation, oxLDL/LOX-1 receptor activation has been shown to elicit the upregulation of signaling pathways that can impact vascular reactivity. (Blair et al., 1999; Cominacini et al., 2001; Ryoo et al., 2006; Ryoo et al., 2011; Xu et al., 2007; Li and Mehta, 2000; Ou et al., 1985; Li and Renier, 2009). For example, oxLDL/LOX-1 receptor activation can contribute to endothelin-1-mediated vasoconstriction. Binding of oxLDL to LOX-1 can also trigger the impairment of endothelial dependent relaxation through decreased nitric oxide (NO) bioavailability and increased reactive oxygen species production [Reviewed by Pirillo et al. (2013)] Ryoo et al. (2006) and Gradinaru et al. (2015). NO is crucial for maintaining vascular tone, and reduction in NO production and/or bioavailability leads to impaired vascular relaxation and contributes to increased vascular resistance leading to endothelial dysfunction (Ryoo et al., 2011; Jiang et al., 2001; Pandey et al., 2014; Tanaka et al., 2009). Together this indicates a critical link between oxLDL, and possibly LOX-1, and vascular pathologies (Ryoo et al., 2011; Jiang et al., 2001; Pandey et al., 2014; Tanaka et al., 2009) which can in turn predispose vessels to structural wall modifications (Ross, 1993). These cascades of events are central to the development of vascular pathologies such as atherosclerosis, aortic aneurysms and the involvement in the acute response to ischemic injury such as stroke.

The oxLDL/LOX-1 pathway plays a complex multimodal role in regulating not only vascular tone and reactivity, but vessel wall integrity and structure. LOX-1 deficiency has been associated with the thinning of adventitial collagen, potentially contributing to increased susceptibility to ruptured aortic aneurysms (Takahashi et al., 2023). It has also been found that LOX-1 plays a key role in the proliferation of cardiac fibroblasts leading to cardiac remodeling (Liu et al., 2016), which has concomitantly been suggested to occur within the vasculature as oxLDL contributes to endothelial dysfunction (Galle et al., 2006). Although these studies provide evidence that LOX-1 plays a pivotal role in altering vessel structural integrity, they underscore the need for further investigation into the mechanisms driving aortic reactivity, stiffness, and remodeling in response to oxLDL-mediated LOX-1 signaling during both physiological and pathological conditions.

OxLDL induced endothelial dysfunction can be precipitated by factors including diet, biological sex, and aging. OxLDL/LOX-1 activation leads to increased oxidative stress and inflammation, significant contributors to vascular dysfunction and aging making LOX-1 a potential therapeutic strategy for the prevention and treatment of CVD (Barreto et al., 2021; Munno et al., 2024). Although fewer studies have examined the influence of sex on LOX-1 expression and regulation under physiological and pathological conditions compared to aging studies (Ogola et al., 2022), its posited that vascular oxLDL/LOX-1 levels or activity may be affected by biological sex. Long-standing studies have reported that estrogen exerts a protective effect against oxidative stress [Reviewed by Khalil (2005)] which could potentially explain the lower oxLDL levels in premenopausal women compared to men. However, as women transition into menopause and ovarian production of 17-beta estradiol decreases, their oxLDL levels tend to increase (Paik et al., 2013) contributing to diminished cardiovascular protective advantages they once enjoyed during the premenopausal years. In terms of vascular reactivity, sex differences in endothelium-dependent responses have been thoroughly investigated [Reviewed by Costa et al. (2023)] Kauser and Rubanyi (1995); Riedel et al. (2019). Endogenous estrogen levels contribute to mechanisms linked to vasodilation (e.g., enhanced NO and prostacyclin production) while androgens are more linked to augmented vasoconstrictor pathways (e.g., enhanced thromboxane synthase activity) (Li et al., 2004) (Krause et al., 2011; Gonzales et al., 2005). Interestingly, at the molecular level, a candidate gene study revealed sex-specific association between the *OLR1* gene, the gene that codes for LOX-1, and carotid plaque formation (Wang et al., 2011). A study by Akhmedov et al. (2014) reported that overexpression of endothelial LOX-1 increases plaque formation and atherosclerosis in male mice. This, combined with other findings demonstrating higher LOX-1 expression in male aortas compared to female aortas (Kim and Hamblin, 2016), suggests that oxLDL/LOX-1 signaling may be influenced by biological sex. However, what specific role oxLDL/LOX-1 plays on vascular function including the consideration of sex-specific responses remains an area of investigation. Therefore, in this study we addressed whether oxLDL alters vascular reactivity, stiffness, and remodeling across age and sex in thoracic aortic ring preparations isolated from gonadally intact 7 and 12 month old male and female mice and determined whether oxLDL mediated actions are LOX-1 dependent.

## 2 Materials and methods

### 2.1 Mice

All mice experiments were approved by the Institutional Animal Care and Use Committee at the University of Arizona (IACUC 16-079, PI: RJG). Male and female mice (C57BL/6J; Jackson Laboratory Bar Harbor, ME) were non-apolipoprotein-E deficient mice to minimize confounding variable of prior exposure to elevated serum levels of oxLDL (4.5 ng/dL) previously found to develop in apolipoprotein-E deficient mice by 10 weeks of age (Kato et al., 2009).

### 2.2 Vasoreactivity assays

Thoracic aortas were isolated from 7-month (n = 19/male; n = 14/female) and 12-month (n = 15/male; n = 18/female) C57BL/6J intact female (not cycled) and male mice. Thoracic aortas were placed in ice‐cold PSS bicarbonate buffer (PSS: 118.99 mM NaCl, 4.69 mM KCl, 1.17 mM MgSO_4_*7H_2_O, 1.18 mM KH_2_PO_4_, 1.60 mM CaCl_2_-2H_2_O, 25.00 mM NaHCO_3_, 0.03 mM EDTA, 5.50 mM glucose) bubbled continuously with 21% O_2_ and 5% CO_2_ and cleaned of fat and connective tissue and cut into 1 mm rings for isometric contractile force measurements. Next, endothelium-intact or -denuded rings were mounted on tungsten wires and immersed in PSS at 37°C with constant gassing (21% O_2_ and 5% CO_2_) in a wire myography chamber (DMT 610) and incubated *ex vivo* with oxLDL (50 μ g/dL; 2 h; Kalen Biomedical, cat. no. 770202–7), followed by normalization and KCL (100 mM) wakeup. Cumulative concentration-response curves to phenylephrine (PE) were generated to determine the contractile response. From these curves the EC50 was determined and used to contract rings to assess acetylcholine (ACh) dependent relaxation. BI-0115 (10 μ M; selective LOX-1 inhibitor; Boehringer Ingelheim) or vehicle (>0.1% dimethyl sulfoxide; DMSO) were given 0.25 h prior to oxLDL in some experiments to determine LOX-1 receptor dependence.

### 2.3 Passive aortic stiffness and remodeling

Thoracic aortic stiffness was determined as previously described by assessing the slope of the stress-strain curve (Del Campo et al., 2019). Incremental recordings of both the force tension and internal diameter were taken during mechanical aortic ring stretching within the tissue bath. The force measurements were recorded by a force transducer and plotted against the corresponding diameter of the aortic rings. Once plotted, a linear regression analysis was performed for each treatment group to ascertain the slope of force over diameter. As described by Del Campo et al. (2019), an increased slope value of the tension over diameter relationship corresponds to an increased stiffness of the aortic ring segment. The remodeling endpoint was determined using the second extrapolated value from the linear regression analysis of the tension over diameter plots in combination with the LaPlace equation. Del Campo et al. (2019) previously described this methodology of estimating the vessel diameter, from which decreased vessel diameter at 100 mmHg would indicate inward remodeling. With regards to the specific protocol, diameter–tension relationships were determined following 2 h incubation, but prior to KCL wakeup by a stepwise stretching of the tissue increasing its passive diameter by increasing the distance between the tungsten wires that were passed through the lumen of the aortic segment. Both the force and internal circumference of each aortic vessel segment was recorded and transformed into vessel diameter. The estimated diameter at 100 mmHg of pressure was determined utilizing the DMT normalization module (LabChart software, ADInstruments) which applies the obtained diameter-tension relationship and Laplace equation. Linear regression of each diameter-tension relationship was utilized to determine passive aortic stiffness and remodeling, corresponding to the steepness of the slope and diameter of vessel at zero pressure in relation to estimated diameter at 100 mmHg. Stress strain curves were also generated to visualize the mechanical elasticity and stiffness changes within the isolated aortic segments from each experimental/drug treatment cohort. Stress was expressed at force (mN)/area (mm^2^) and strain calculated as the ratio of (initial diameter–final diameter)/final diameter.

### 2.4 Quantitative real time PCR

To investigate potential molecular mechanisms underlying oxLDL-mediated changes in vasoreactivity, stiffness, and remodeling during aging, we examined the levels of relevant mediators in thoracic aortas from 1 month old and a small cohort of 16 month old aged male mice. Quantitative real time PCR (qRT-PCR) was utilized to measure changes in mRNA levels of *LOX-1*, *CD36*, *ET-1*, *ET-1R* α *, ET-1R* β , and *IL-6* in isolated 1 month and 16 month murine male thoracic aortas as previously described (Wendt and Gonzales, 2023). Primer sequences described in Table 1.

**TABLE 1:** qRT-PCR primers.

Primer	Sense (5′-3′)	Anti-Sense (5′-3′)
LOX-1	GCTCTGCTTCTCGTGGGCAT	CGAAGGCCCCAAGGAAAGGG
CD36	TGTGGCAAACAGGGCTGGAG	GCAAGCACAAGTCTGGATCACC
ET-1	ACGCCAGTGCTAATGGCTCC	AGGTGTCTGCACTCAAGGCG
ET-1Rα	TGCTTTGATCAGGCACCCTCC	CCCAGAGCTGACTTCTGCCG
ET-1Rβ	ATGCCCTGATGCCTTAGCCAC	ACCCACCTGCAGAGCAAGAAC
IL-6	CCAAGAGGTGAGTGCTTCCCC	ACTCTCTCCCTTCTGAGCAGC

### 2.5 Reagents

All reagents were purchased from Sigma Aldrich (St. Louis, MO) unless otherwise noted.

### 2.6 Data and statistical analysis

For each treatment group, an n ≥ 3 was used to achieve an acceptable power for statistical analysis. Experiments were repeated for statistical analysis and data graphed using Prism 9.3.0 for Windows, GraphPad Software, United States, www.graphpad.com (GraphPad Software). Shapiro-Wilk and F-tests were performed to confirm if data sets achieved a normal distribution and equal variance among groups. Direct comparisons between two groups were made using an unpaired *t*-test. Comparisons between three or more groups were made using a two-way ANOVA with Tukey’s multiple comparisons *post hoc* test. *p* < 0.05 was considered significant. Values are expressed as means ± SEM.

## 3 Results

### 3.1 PE-induced contractile responses are sex and age dependent in murine thoracic aortic ring segments

**FIGURE 1:**
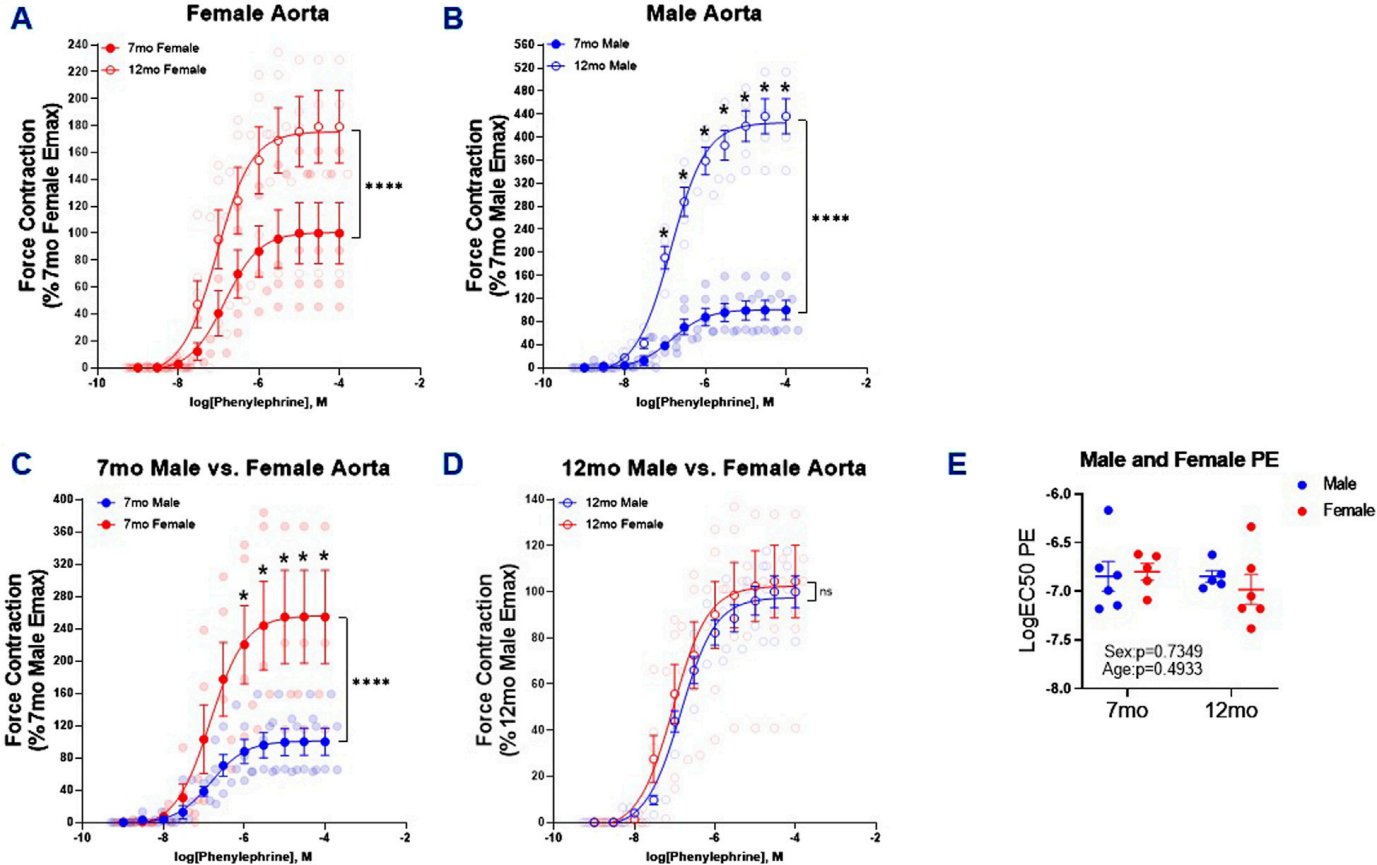
PE-Induced Contraction of Thoracic Aortic Rings. Concentration response curves **(A–D)** to PE in 1 mm thoracic aortic rings from 7- and 12-month-old male and female mice. Graph in figure **(E)** represents LogEC50 of PE obtained from concentration response curves in male 7- (n = 6) and 12 month (n = 5) and female 7- (n = 4–5) and 12-month-old (n = 6) mice. Individual values are shown are transparent data points. Grouped data are represented as means ± SEM. Two-Way ANOVA with Tukey’s *post hoc* test *p < 0.05, ****p < 0.0001.

It has been previously shown that 26 month male C57BL/6 mice exhibited increased aortic contraction compared to younger 5 month mice in response to PE (De Moudt et al., 2022). Similarly, in this study PE-induced thoracic aortic contraction was increased in both 12 month female and male mice relative to 7 month counterparts (Figures 1A, B); and this increase was most pronounced in the males (×4.4) as compared to the females (×1.8). When comparing males to females there was a notable sex dependent increase in the efficacy of PE-induced contraction in the females compared to males at 7-month-old (Figure 1C). That was not observed in 12-month-old mice (Figure 1D). While there were notable effects in age and sex on PE-induced contraction efficacy, we did not observe alterations to the potency of PE (Figure 1E).

### 3.2 ACh-induced relaxation responses are sex and age dependent in murine thoracic aortic ring segments

**FIGURE 2:**
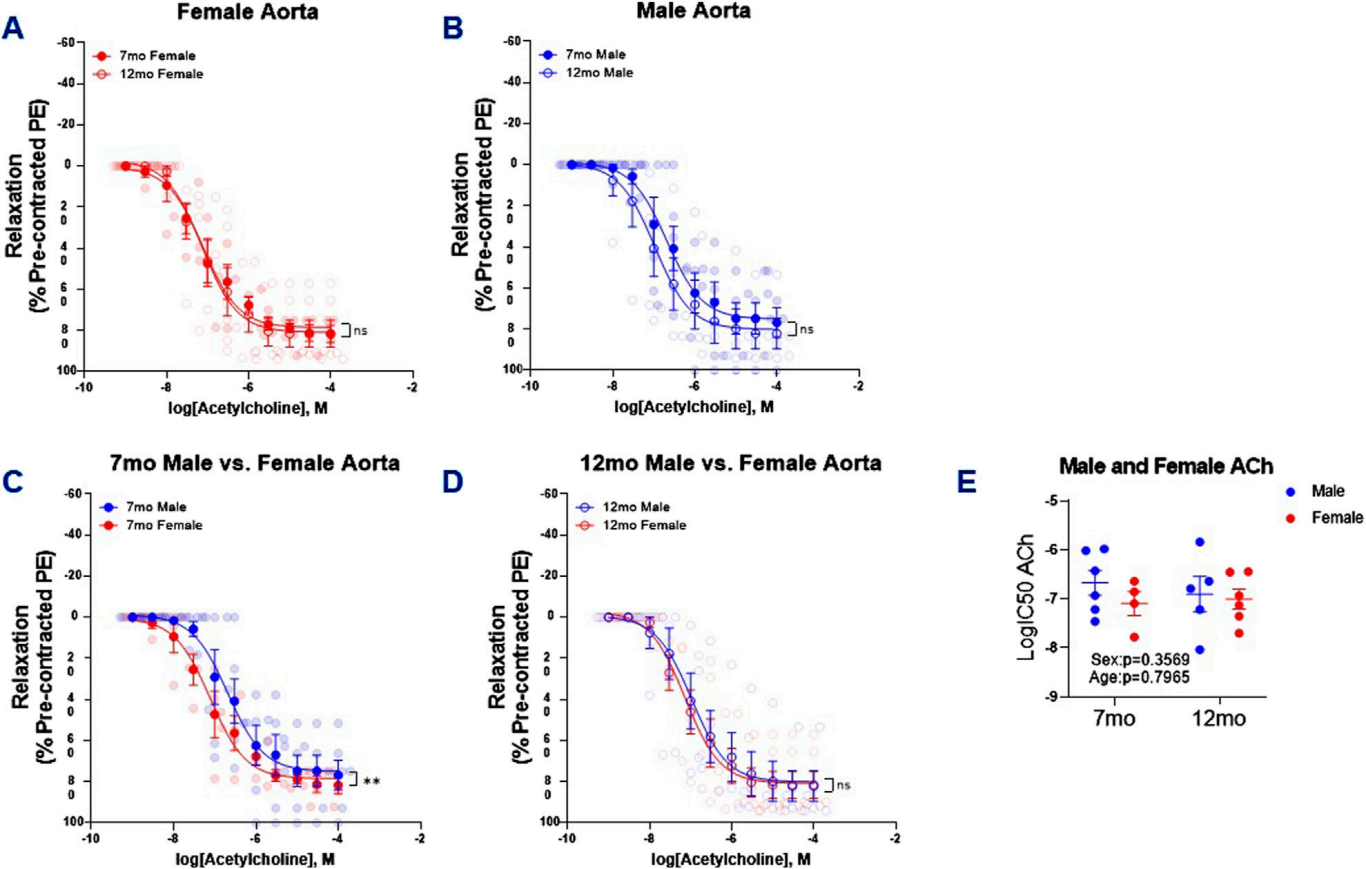
ACh-Induced Relaxation PE Pre-Contracted Thoracic Aortic Rings. Concentration response curves **(A–D)** to ACh (PE-precontracted) in 1 mm thoracic aortic rings from 7- and 12-month-old male and female mice. Figure **(E)** illustrates LogIC50 of ACh obtained from concentration response curves in male 7- (n = 6) and 12 month (n = 5) and female 7- (n = 4–5) and 12-month-old (n = 6) mice. Individual values are shown are transparent data points. Grouped data are represented as means ± SEM. Two-Way ANOVA with Tukey’s *post hoc* test **p < 0.01.

From the PE cumulative concentration-response curves we derived the EC50 for each animal to next assess ACh-mediated relaxation. Counter to our observations of age-dependent differences in PE-induced aortic contraction, there were no differences in 7 and 12 month female (Figure 2A) and 7 and 12 month male ACh mediated relaxation (Figure 2B). However, in alignment with previous observations made in 5 month male and female Sprague–Dawley rats (Akther et al., 2021), ACh-induced relaxation was greater in 7 month female mice than males (Figure 2C). This sex difference in response to ACh was diminished in 12 month male and female mice (Figure 2D). Similar to PE-induced contraction of the thoracic aortas, there was no changes in the potency of ACh-mediated relaxation in the 7 and 12 month male and female mice (Figure 2E).

### 3.3 OxLDL increased PE-induced contraction in male thoracic aortic rings in a LOX-1, age, and endothelial dependent manner

**FIGURE 3:**
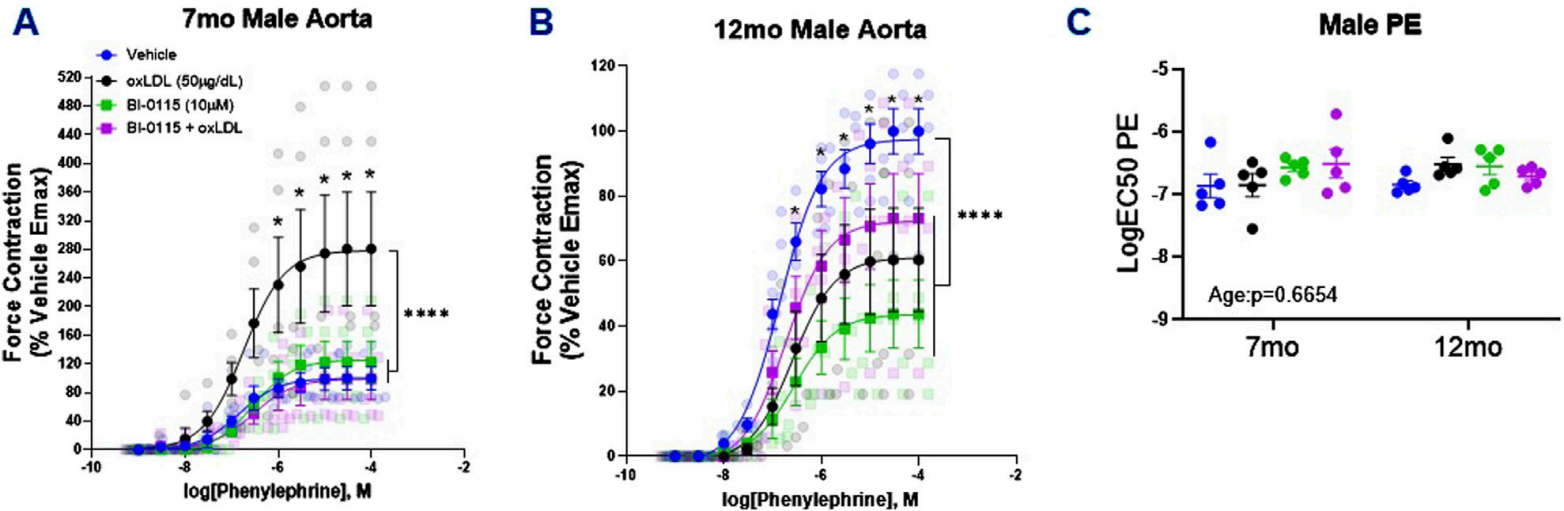
Male Thoracic Aortic Ring Contraction of 7- and 12-Month-Old Mice Following oxLDL Exposure, LOX-1 Inhibition, and oxLDL + LOX-1 Inhibition. Concentration response curves **(A, B)** to PE in 1 mm thoracic aortic rings from 7- and 12-month-old male mice following exposure to either vehicle (7 month: n = 5–6; 12 month: n = 5), oxLDL (50 μ g/dL) (7 month: n = 5–6; 12 mo.:n = 5), BI-0115 (10 μ M; selective LOX-1 inhibitor) (7 month: n = 5–7; 12 month: n = 5), or oxLDL (50 μ g/dL) + BI-0115 (10 μ M) (7 month: n = 5–7; 12 month: n = 5) for 2 h. In aortas exposed to BI-0115, the selective inhibitor was 12-month-old administered 0.25 h prior to oxLDL exposure. **(C)** Graph representing LogEC50 of PE obtained from concentration response curves in 7- and 12-month-old male mice exposed as previously described. Individual values are shown are transparent data points. Grouped data are represented as means ± SEM. Two-Way ANOVA with Tukey’s *post hoc* test *p < 0.05, ****p < 0.0001.

We next assessed the impact of acute oxLDL (50 μ g/dL; 2 h) exposure on 7 month and 12 month male thoracic aortic PE-induced contraction. We observed a prominent increase in the efficacy of PE-induced contraction in 7 month male thoracic aortas incubated with oxLDL for 2 h compared to vehicle (Figure 3A). This oxLDL-mediated increase in PE contraction was attenuated with selective LOX-1 inhibition (BI-0115; 10 μ M), suggesting that LOX-1 mediates the observed oxLDL increase in PE-induced contraction in 7 month old male thoracic aortic. When examining denuded ring preparations from 7-month-old, we observed that denudation greatly abrogated the oxLDL-mediated increase in PE efficacy that was observed in endothelial intact aortas and that LOX-1 inhibition alone or in the presence of oxLDL significantly attenuated the PE contractile response (Supplementary Figure S1A). Together these data suggest that the increased efficacy of PE-induced contraction in the 7 month male mice is mediated via endothelial LOX-1 dependence. Intriguingly, upon examination of the oxLDL/LOX-1 axis within 12 month male thoracic aortic rings, we observed a significant decrease in the efficacy of PE-mediated contraction in rings incubated with oxLDL, LOX-1 inhibition, and the combination of oxLDL plus LOX-1 inhibition (Figure 3B). However, we did not observe a change in the potency of PE-mediated contraction regardless of age or treatment (Figure 3C). Thus, together these data suggest that alterations in PE induced contraction by the oxLDL/LOX-1 axis in male mice is LOX-1, age, and endothelial dependent.

### 3.4 OxLDL attenuated ACh-induced relaxation in male thoracic aortic rings in an age and endothelial dependent manner

**FIGURE 4:**
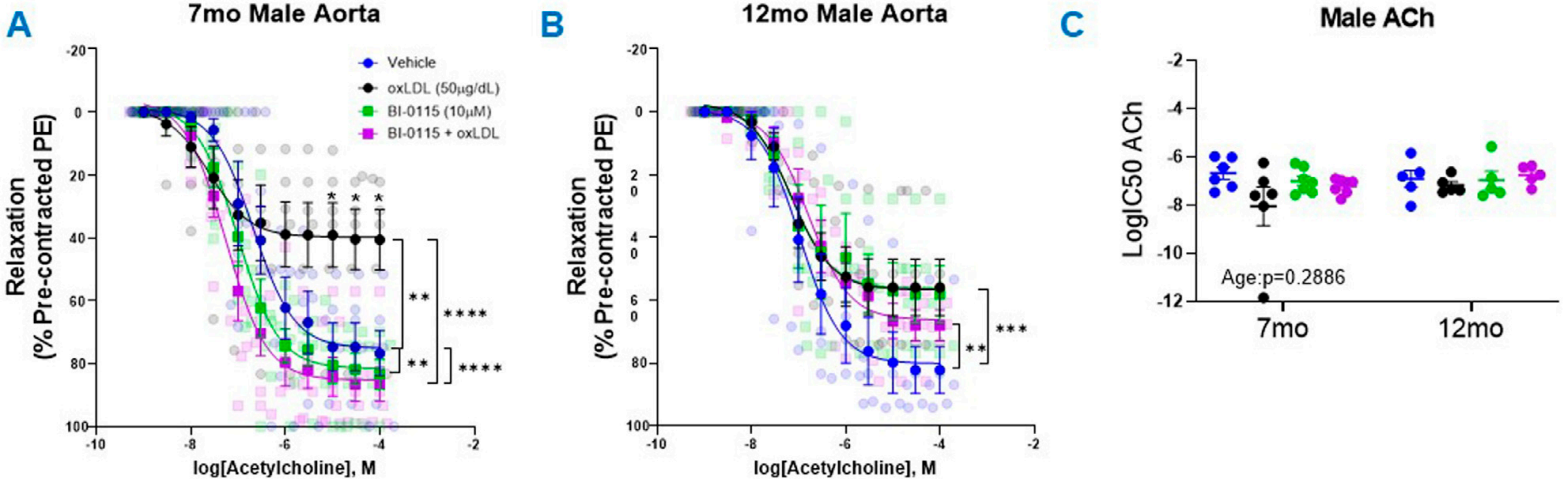
Male Thoracic Aortic Ring Relaxation of 7- and 12-Month-Old Mice Following oxLDL Exposure, LOX-1 Inhibition, and oxLDL + LOX-1 Inhibition. Concentration response curves **(A, B)** to ACh (PE-precontracted) in 1 mm thoracic aortic rings from 7- and 12-month-old male mice following exposure to either vehicle (7 month: n = 5–6; 12 month: n = 5), oxLDL (50 μ g/dL) (7 month: n = 5–6; 12 month: n = 5), BI-0115 (10 μ M; selective LOX-1 inhibitor) (7 month: n = 5–7; 12 month: n = 5), or oxLDL (50 μ g/dL) + BI-0115 (10 μ M) (7 month: n = 5–7; 12 month: n = 5) for 2 h. In aortas exposed to BI-0115, the selective inhibitor was administered 0.25 h prior to oxLDL exposure. **(C)** Graph representing LogIC50 of ACh obtained from concentration response curves in 7- and 12-month-old male mice exposed as previously described. Individual values are shown are transparent data points. Grouped data are represented as means ± SEM. Two-Way ANOVA with Tukey’s *post hoc* test *p < 0.05, **p < 0.01, ****p < 0.0001.

Following the PE-induced contractility studies, we next assessed the effects of oxLDL/LOX-1 on ACh-mediated relaxation in 7 month and 12 month aortic rings pre-contracted with PE (predetermined EC50 dose) (Figure 4). In both the 7 month (Figure 4A) and 12 month (Figure 4B) male thoracic aortic rings, incubation with oxLDL *ex vivo* significantly attenuated ACh-mediated relaxation compared to vehicle treated rings. We further observed that LOX-1 inhibition with BI-0115 (LOX-1 inhibitor) attenuated this response in the rings collected from 7 month old male mice however BI-0115 had no effect on ACh-mediated relaxation in oxLDL treated aortic rings from 12 month old male mice suggesting an age dependent response to ACh relaxation following exposure oxLDL. When the aortic rings were denuded, ACh-mediated relaxation was nearly abolished and the effect of oxLDL nullified when compared to vehicle (Supplementary Figure S1B). We further observed that BI-0115 alone and BI -0115 plus oxLDL elicited a nominal increase in contraction in response to concentration response curve to ACh perhaps suggesting a smooth muscle LOX-1 dependent contractile response independent of oxLDL (Supplementary Figure S1B **)**. Although changes in the efficacy of the ACh response curve following either oxLDL, BI-0115 alone, or oxLDL plus BI-0115 were observed, there were no changes in the potency of ACh-mediated relaxation (Figure 4C). Together, these data suggest that although oxLDL attenuates endothelial dependent relaxation, involvement of LOX-1 activation is age dependent between aortas from both 7- and 12-month-old male mice.

### 3.5 Female thoracic aortic PE-induced contraction was decreased following oxLDL exposure in an endothelial dependent manner, and independent of LOX-1

**FIGURE 5:**
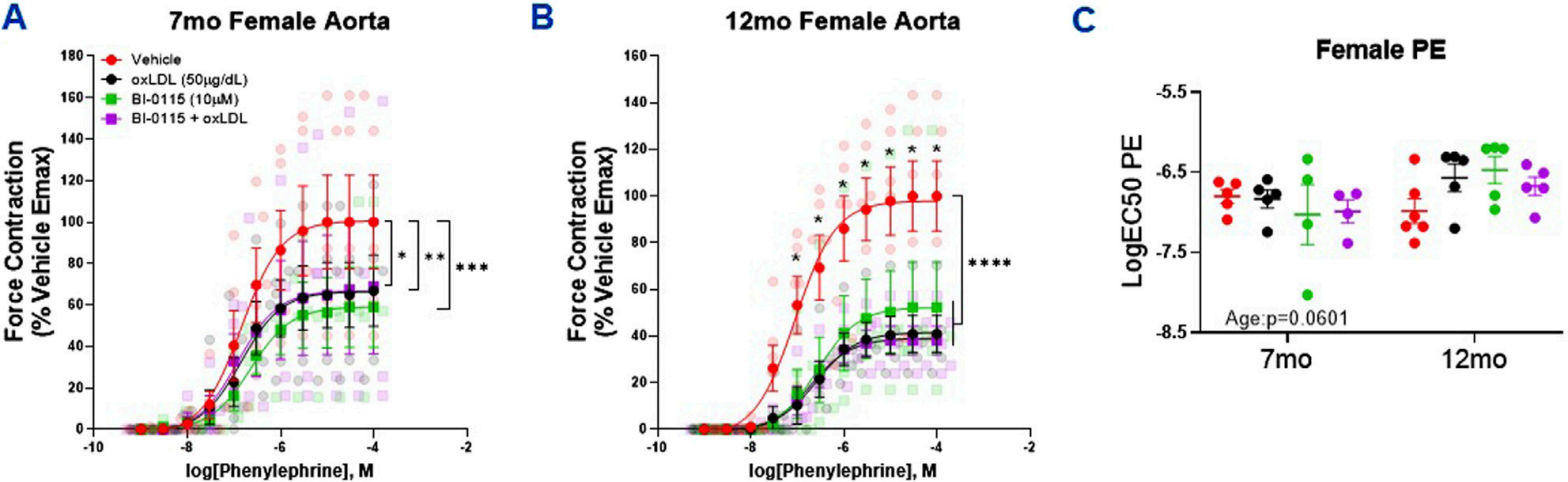
Female Thoracic Aortic Ring Contraction of 7- and 12-Month-Old Mice Following oxLDL Exposure, LOX-1 Inhibition, and oxLDL + LOX-1 Inhibition. Concentration response curves to **(A, B)** PE in 1 mm thoracic aortic rings from 7- and 12-month-old female mice following exposure to either vehicle (7 month: n = 4–5; 12 month: n = 5), oxLDL (50 μ g/dL) (7 month: n = 4–5; 12 month: n = 6), BI-0115 (10 μ M; selective LOX-1 inhibitor) (7 month: n = 3–4; 12 month: n = 5–6), or oxLDL (50 μ g/dL) + BI-0115 (10 μ M) (7 month: n = 3–4; 12 month: n = 5–6) for 2 h. In aortas exposed to BI-0115, the selective inhibitor was administered 0.25 h prior to oxLDL exposure. **(C)** Graph representing LogEC50 of PE obtained from concentration response curves in 7- and 12-month-old male mice exposed as previously described. Individual values are shown are transparent data points. Grouped data are represented as means ± SEM. Two-Way ANOVA with Tukey’s *post hoc* test *p < 0.05, **p < 0.01, ***p < 0.001, ****p < 0.0001.

We first assessed thoracic aortic contractility via PE in both 7 and 12 month old. females and observed a decrease in contractility following exposure to either oxLDL, LOX-1 inhibition, or oxLDL plus LOX-1 inhibition (Figures 5A, B). These results are similar to our previous findings in 12-month-old male thoracic aortas; however, they contrast with the contractility responses observed in 7-month-old male aortas. When aortic rings were denuded, as observed previously in our male denuded preparations (Supplementary Figure S1A), the effect of oxLDL was nullified in denuded female aortic rings (Supplementary Figure S1C). And similar to that observed in males, we did not observe a change in the potency of PE-mediated contraction in females regardless of age or treatment (Figure 5C).

### 3.6 Female thoracic aortic ACh-induced relaxation was differentially altered in a LOX-1, age, and endothelial dependent manner

**FIGURE 6:**
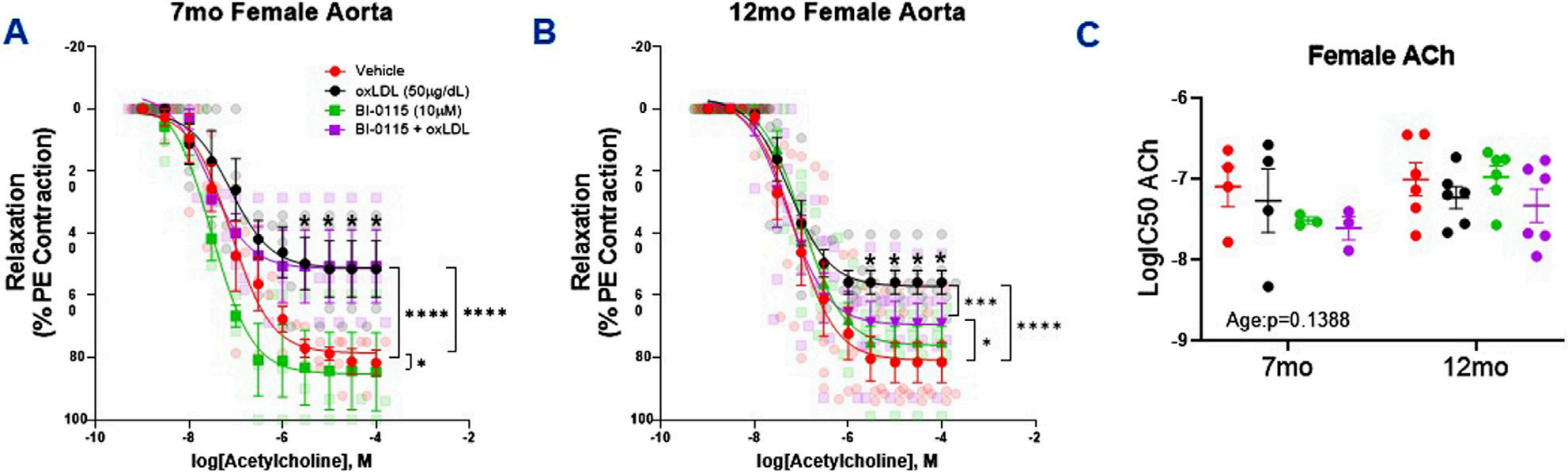
Female Thoracic Aortic Ring Relaxation of 7- and 12-Month-Old Mice Following oxLDL Exposure, LOX-1 Inhibition, and oxLDL + LOX-1 Inhibition. Concentration response curves **(A, B)** to ACh (PE-precontracted) in 1 mm thoracic aortic rings from 7- and 12-month-old female mice following exposure to either vehicle (7 month: n = 4–5; 12 month: n = 5), oxLDL (50 μ g/dL) (7 month: n = 4–5; 12 month: n = 6), BI-0115 (10 μ M; selective LOX-1 inhibitor) (7 month: n = 3–4; 12 month: n = 5–6), or oxLDL (50 μ g/dL) + BI-0115 (10 μ M) (7 month: n = 3–4; 12 month: n = 5–6) for 2 h. In aortas exposed to BI-0115, the selective inhibitor was administered 0.25 h prior to oxLDL exposure. **(C)** Graph representing LogIC50 of ACh obtained from concentration response curves in 7- and 12-month-old male mice exposed as previously described. Individual values are shown are transparent data points. Grouped data are represented as means ± SEM. Two-Way ANOVA with Tukey’s *post hoc* test *p < 0.05, ***p < 0.001, ****p < 0.0001.

When assessing the impact of oxLDL on endothelial dependent relaxation in the female mice, we observed that oxLDL attenuated ACh induced relaxation in both 7- and 12-month-old female aortic rings (Figures 6A, B). This attenuative oxLDL response on the ACh induced relaxation appeared to be less prominent in both the 7 month (51.5 ± 9.1% relaxation) and 12 month (55.8 ± 3.7% relaxation) female aortas visually compared to that observed in the 7 month male aortas (40.6 ± 9.5% relaxation) but more comparable to the 12 month males (55.94 ± 9.0% relaxation). Interestingly, similar to 7 month male aortic rings, BI-0115 (LOX-1 inhibitor) alone further enhanced endothelial relaxation in the 7 month females (Figure 6A) but not in the 12 month female mice (Figure 6B). Moreover, oxLDL-mediated alteration in ACh relaxation in the 12 month females was slightly attenuated by LOX-1 inhibition (Figure 6B), which was not observed in the 7 month females (Figure 6A). These results directly oppose the observations in the male mice (Figures 4A, B). As observed previously, while oxLDL, LOX-1 inhibition, and oxLDL plus LOX-1 inhibition altered ACh efficacy, there was no change in potency of ACh (Figure 6C). In denuded preparations, similar to males, ACh-mediated relaxation was abolished and the effect of oxLDL nullified when compared to vehicle (Supplementary Figure S1D). When taken together, in females, these data suggest that 1) acute oxLDL exposure attenuates ACh-induced relaxation of 7 and 12 month endothelial intact aortic rings, 2) oxLDL-induced ACh relaxation is partially LOX-1 dependent in the 12 month females but not in the 7 month females, and finally, 3) BI-0115 treatment alone further enhances ACh-induced relaxation in the 7 month female thoracic aortas similar to that observed in the age matched males.

### 3.7 OxLDL increases thoracic aortic stiffness in an age, sex, endothelial, and LOX-1 dependent manner

**FIGURE 7:**
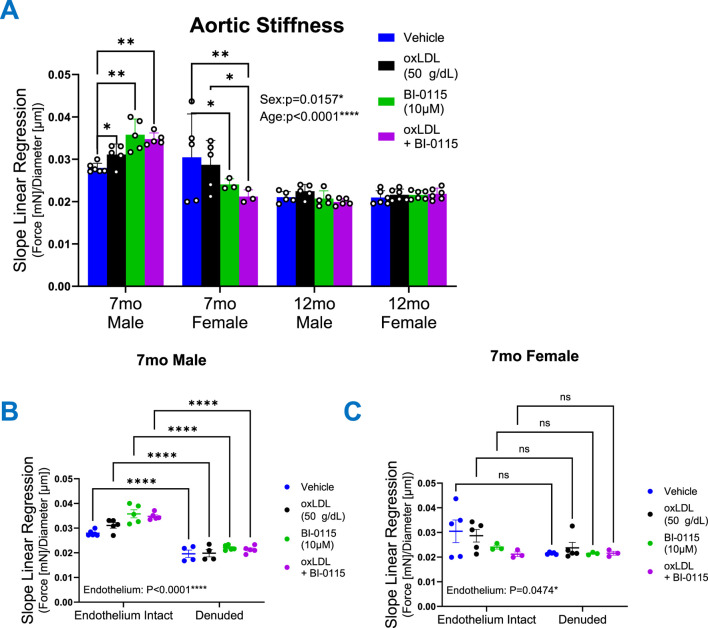
OxLDL Differentially Alters Thoracic Aortic Stiffness. Graphs depicting wire myography mediated **(A–C)** linear regression analysis of diameter–tension relationships from 1 mm thoracic aortic vessels obtained from 7- and 12-month-old male and female mice following exposure to either vehicle (Male: 7 month: n = 6; 12 month: n = 5; Female: 7 month: n = 4–5; 12 month: n = 6), oxLDL (50 μ g/dL) (Male: 7 month: n = 5; 12 month: n = 5; Female: 7 month: n = 4–5; 12 month: n = 6), BI-0115 (10 μ M; selective LOX-1 inhibitor) (Male: 7 month: n = 5; 12 month: n = 5; Female: 7 month: n = 3; 12 month: n = 6), or oxLDL (50 μ g/dL) + BI-0115 (10 μ M) (Male:7 month: n = 5; 12 month: n = 5; Female: 7 month: n = 3; 12 month: n = 6) for 2 h. Data are represented as means ± SEM. Two-Way ANOVA with Tukey’s *post hoc* test *p < 0.05, **p < 0.01, ***p < 0.001, ****p < 0.0001.

We next examined how acute oxLDL exposure impacts the thoracic aorta functionally in terms of stiffness (Figure 7). Utilizing the diameter-tension curves from both 7- and 12-month-old male and female aortic ring studies, calculated measurements resulted in an overall effect of both sex and age on stiffness (Figure 7A). These data indicate that 7 month thoracic aortic rings were stiffer in comparison to 12 month rings and that male mice had increased stiffness compared to females. Additionally, oxLDL as well as the selective LOX-1 inhibitor, BI-0115, in the presence or absence of oxLDL increased stiffness in 7 month male aortas in an endothelial dependent manner (Figure 7B). Whereas oxLDL did not influence the stiffness of 12 month males, or 7- and 12-month-old females. Interestingly, the selective LOX-1 inhibitor alone or in the presence of oxLDL decreased 7 month female aortic stiffness however this response was not endothelial dependent (Figure 7C). While there was an overall effect of age on aortic stiffness, we did not observe an effect of oxLDL, LOX-1 inhibition, or oxLDL plus LOX-1 inhibition on 12 month male and female aortic stiffness (Figure 7A). Accompanying stress strain curves are included in Supplementary Figures S3A–D (7-month-old male and female endothelium intact and denuded) and in Supplementary Figures S4A, B (12-month-old male and female endothelium intact only).

### 3.8 OxLDL increases aortic remodeling in a sex, endothelial, and LOX-1 dependent manner

**FIGURE 8:**
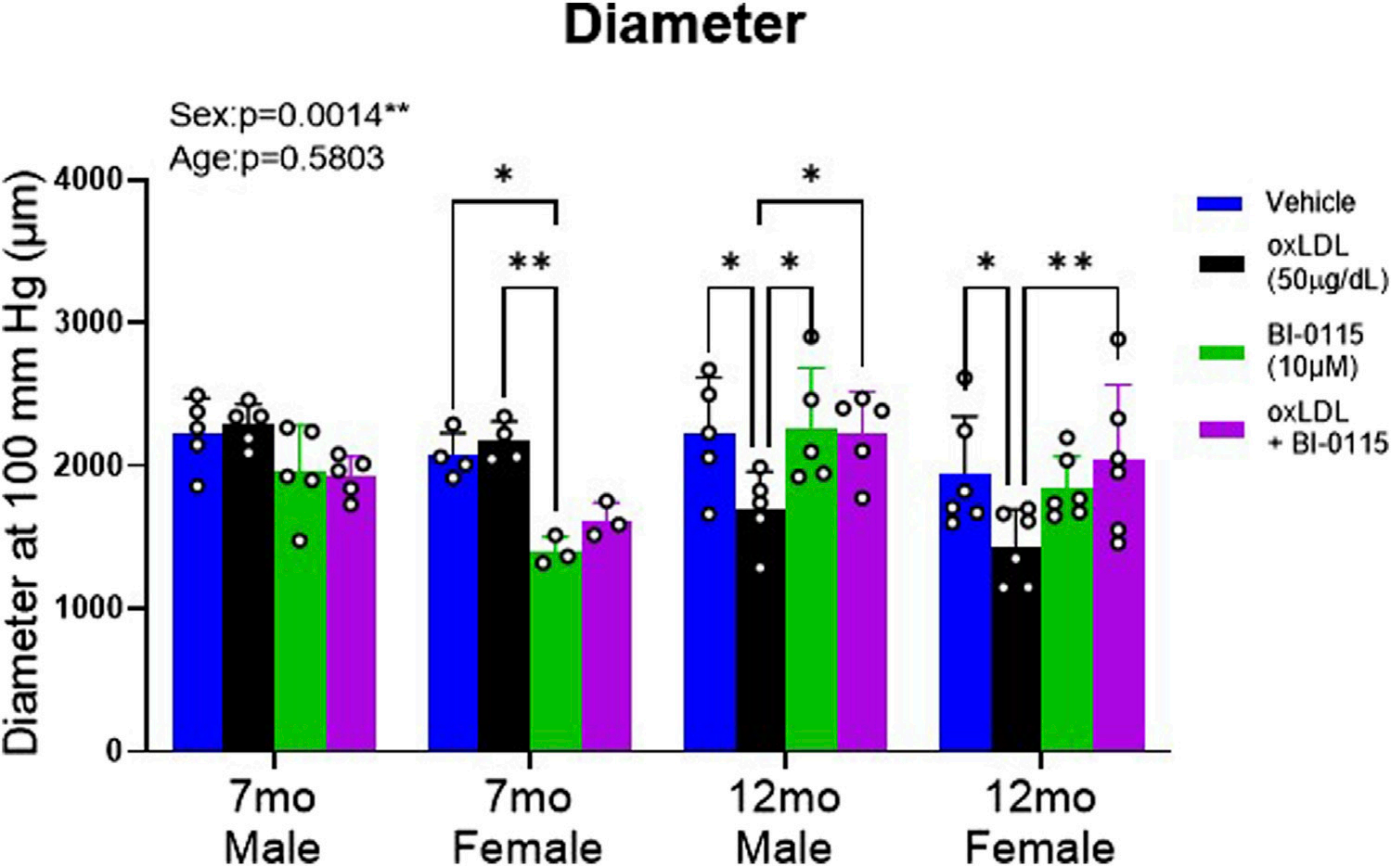
OxLDL Effect Remodeling in Thoracic Aortic Rings in a Sex, Endothelial, and LOX-1 Dependent Manner. Estimated diameter at 100 mmHg from 1 mm thoracic aortic rings was calculated in 7- and 12-month-old male and female mice to predict inward remodeling following exposure to either vehicle (Male: 7 month: n = 6; 12 month: n = 5; Female: 7 month: n = 4–5; 12 month: n = 6), oxLDL (50 μ g/dL) (Male: 7 month: n = 5; 12 month: n = 5; Female: 7 mo: n = 4–5; 12 month: n = 6), BI-0115 (10 μ M; selective LOX-1 inhibitor) (Male: 7 month: n = 5; 12 month: n = 5; Female: 7 month: n = 3; 12 month: n = 6), or oxLDL (50 μ g/dL) + BI-0115 (10 μ M) (Male: 7 month: n = 5; 12 month: n = 5; Female: 7 month: n = 3; 12 month: n = 6) for 2 h *p < 0.05, **p < 0.01.

By collecting aortic ring force and internal circumference measurements, we transformed these data recordings to calculate vessel diameter (see Section 2 for details). In terms of the finding, although we did not observe an overall effect of aging 5 months in mice on inward aortic remodeling we did however, observe a significant impact of sex suggesting that female thoracic aortas are smaller in diameter compared to males (Figure 8). Upon further examination addressing the effects of oxLDL, dissimilar to our observations of altered stiffness, we did not observe an effect of oxLDL on 7 month male and female remodeling. However, we did observe that in denuded 7 month male aortic rings, oxLDL, BI-0115, and oxLDL plus BI-0115 increased inward remodeling (Supplementary Figure S2A). We also observed that endothelial removal resulted in inward remodeling of 7 month female aortic rings (Supplementary Figure S2B). The LOX-1 inhibitor in plus or minus oxLDL had no effect. In the older cohorts, oxLDL induced a significant inward remodeling in both endothelial intact 12 month male and female aortic rings, and these responses were LOX-1 dependent (Figure 8). From this we hypothesized that perhaps 12 month mice may be potentially metabolizing or up taking oxLDL at a greater rate compared to the 7-month-old mice resulting in a smaller diameter at 100 mmHg. In efforts to elucidate potential molecular mechanisms of oxLDL mediated altered vasoreactivity, stiffness, and remodeling in aging, we assessed levels of possible involved mediators within the thoracic aortas from 1-month-old and a small cohort of 16-month-old aged male mice (Supplementary Figure S5). We observed that oxLDL increased mRNA levels of LOX-1, endothelin-1, endothelin-1-receptors alpha and beta, and interleukin-6. Intriguingly however, we did not observe an increase in CD36 expression following oxLDL exposure but did observe an increase in CD36 expression with aging. Moreover, we also observed that levels of LOX-1, endothelin-1-receptor beta, and interleukin-6 were increased with age, but not endothelin-1 and endothelin-1-receptor alpha. Together these data suggest that increased age may potentiate a preferential increase in both LOX-1 and CD36-mediated uptake of oxLDL, and future studies will determine if these factors play a role in increased inward remodeling that we observed in our 12-month-old mice.

## 4 Discussion

In the present study we employed an *ex vivo* thoracic aortic ring preparation to evaluate the impact of the oxLDL/LOX-1 axis on vasoreactivity, stiffness, remodeling, and transcription in the context of sex and age to further elucidate the detrimental role oxLDL/LOX-1 plays in altering vascular function and health. Additionally, we evaluated the role of LOX-1 receptor dependance on thoracic aortic vascular reactivity and endothelial function. We have for the first time, demonstrated the influence of sex, age, endothelium dependence, and LOX-1 activation following 2 h *ex vivo* oxLDL exposure in mediated alterations in thoracic aortic vasoreactivity, stiffness, and remodeling. In brief, oxLDL exposure 1) increased contractility of 7 month old male thoracic aortas in response to PE in an endothelial and LOX-1 dependent manner, 2) decreased contractility in 12 month old males as well as 7 and 12 months old females, 3) induced altered ACh dependent relaxation (indirect assessment of endothelial dysfunction) in all cohorts regardless of age or sex, but was most pronounced and LOX-1 dependent in the 7 month old males compared to 12 month old males and 7 and 12 months old females, 4) increased aortic stiffness in 7 month males in a LOX-1 independent manner, and 5) increased inward remodeling of 12 month old male and female thoracic aortas in a LOX-1 dependent manner.

Elucidating the sexual dimorphism in response to cardiovascular pathology such as elevated levels of oxLDL is significant however the impact of oxLDL on vascular reactively has not been thoroughly investigated. Thus, we considered sex as a critical factor in mediating outcomes due to the burgeoning and substantial amount of prior published work demonstrating sex differences during cardiovascular disease. In agreement with other published reports, our data demonstrate that oxLDL via LOX-1 can play a significant role in modulating thoracic aortic vascular reactivity, in part by mediating endothelial dysfunction. Not yet reported by others, our findings suggest that alterations in thoracic aortic vascular reactivity to acute exposure of oxLDL is sex dependent and may potentially contribute to the variations and susceptibility to vascular diseases observed between men and women clinically. Although in this study we did not address vascular LOX-1 expression levels in male verses female, Matilla et al. (2022) demonstrated higher levels of LOX-1 expression cardiac valvular tissues from males verses females. This heightened expression of LOX-1 in males may contribute to a greater predisposition to oxidative stress and inflammation, key drivers of impaired vascular reactivity. In terms of sex differences associated with vascular wall integrity, clinically aortic dissection and rupture have been reported to be higher amongst males compared to females (Olsson et al., 2006). However, this finding has been convoluted by more recent studies demonstrating that thoracic aortic aneurysm mediated death and acute symptoms are higher in women than men (Boczar et al., 2019). Boczar et al. (2019) reported that aneurysmal growth was more than twice as fast in female as it was in male patients and that aortic stiffness was associated with this increase in growth in females but not males. Prior studies examining potential mechanisms underlying these clinical sex differences have found that in experimental aortic aneurysms, female mice exhibited higher levels of estrogen receptor alpha as well as lower matrix metalloproteinase 9 and 2 activity compared to males which could be contributing to increased vasoprotective effects and decreased vascular inflammation in females (Laser et al., 2014). It has also been found in the International Registry of Acute Aortic Dissection, that females presenting with acute aortic dissection were older than males (Nienaber et al., 2004), further suggesting sex differences potentially due to the loss of vasoprotective effects of estrogen upon menopause (Gavin et al., 2009). Understanding these sex differences is crucial for tailoring medical interventions and improving outcomes for both men and women diagnosed with thoracic aortic pathology.

In addition to sex differences, aging is linked to various structural, mechanical, and functional alterations in the vasculature, which are marked by increased arterial stiffness, diminished elasticity, endothelial dysfunction, and changes in vascular tone [Reviewed by Harvey et al. (2016)]. Additionally, the effect of age has been shown to play a role in vascular function at the proteomic and genomic level (Tyrrell et al., 2022; Wan et al., 2023), highlighting the detrimental impact that age plays in the progression and continuation of vascular disease. It has been previously found that aging transforms the human aortic proteome from healthy to a pathological state (thoracic aortic aneurysms), leading to a differential regulation of biological processes (Tyrrell et al., 2022). Several of these processes found to be downregulated with aging include extracellular matrix organization, receptor-mediated endocytosis, and golgi vesicle transport which play all play a role in vasoreactivity and function (Mammoto et al., 2022; Cui et al., 2020; Lee et al., 2009). These proteomic changes may play a crucial role in the deterioration of thoracic aortic function with age. Additionally, a cross-sectional study indicated that the normal aging process within the aorta is associated with gradual aortic elongation and a notable change in aortic geometry (Adriaans et al., 2018). This structural alteration may contribute to age-related changes in aortic function and will be examined in our future studies as it relates to oxLDL/LOX-1. This is further substantiated by a separate study which revealed age-related unfolding of the aortic arch is linked to increased proximal aortic stiffness even in humans free of overt cardiovascular disease, reflecting the interplay of age-related changes that could prompt pathology (Redheuil et al., 2011). Morphology of the human aorta undergoes age-related changes, affecting its diameter, length, and other key aspects such as atherosclerosis (Greenwald, 2007; da Silva et al., 1999; Sawabe et al., 2011; Collins et al., 2014; Fritze et al., 2012; Yamada et al., 2015). These morphological alterations are likely to contribute to the overall decline in thoracic aortic function as the vasculature ages. The aging process is multifaceted and exerts effects on thoracic aortic function through structural changes, alterations in aortic proteome regulation, and modifications in aortic morphology and geometry. Further understanding these mechanisms, especially under pathology such as hyperlipidemia, is crucial for developing targeted interventions to mitigate age-related thoracic aortic dysfunction.

In this study we observed that oxLDL exposure induced endothelial dysfunction resulting in endothelial dependent differentially altered vasoreactivity across a modest span of aging and sex which appear to be in part LOX-1 receptor dependent. It has been previously found that oxLDL mediates an upregulation of endothelin-1, a potent vasoconstrictor, in endothelial cells (Niemann et al., 2005), which corresponds to our findings at the mRNA level (Supplementary Figure S5) and could in part play a role in our observations of increased PE induced contractility in 7 month males (Figure 1). This is further perpetuated by findings demonstrating that blockade of ET-1R α / β improved endothelial-dependent vasodilation (Settergren et al., 2008), which were observed to increase following oxLDL exposure in this study (Supplementary Figure S5). While ET-1 is known to play a role in contractility, a separate study demonstrated that ET-1 is a potent vasoconstrictor in the abdominal aorta; however, in the thoracic aorta a 10 nM dose of ET-1 only induced a 7.8% contraction compared to 60 mM of K^+^ (Zhou et al., 2004).

Intriguingly, we observed a concomitant increase in basal expression of ET-1Rβ in 16 month male thoracic aortas but not alpha. This may suggest an increase in ET-1Rβ mediated increase within the endothelium and subsequential increase in ET-1Rβ -mediated relaxation in the 12 month male mice. In turn, this can result in attenuated contractility in response to PE as well as greater a ACh-mediated relaxation, potentially due to previously demonstrated expression of ET-1Rβ within the endothelium (Khimji and Rockey, 2010; Sakurai et al., 1990; Levin, 1995) and an ET-1Rβ-mediated relaxation in rat thoracic aortic vessels (Tykocki et al., 2009). Expression of endothelin-1 and its receptors merits further investigation across the sexes as it has been previously observed that in human internal mammary arteries, exposure to 17β-estradiol resulted in downregulation of ET-1Rα/β expression (Haas et al., 2007) suggesting that females may respond differentially along the endothelin-1/receptor axis compared to males and may in part delineate the decreased contractility observed in 7 and 12 months female aortic rings exposed to oxLDL.

In addition to increased interest regarding vasoreactivity, there has been a growing interest in elucidating the relationship between oxLDL and aortic stiffness as well as remodeling. Previous work such as the pivotal “Health ABC Study” (Brinkley et al., 2009), has contributed substantially to this line of interest. Wherein the authors explored the correlation between plasma oxLDL levels and arterial stiffness in older adults, revealing via pulse wave velocity measurements a significantly increased incidence of high arterial stiffness with increased oxLDL levels. Moreover, in a separate study, it was demonstrated that LOX-1 was associated with arterial stiffness in both middle-aged and elderly men and women (Otsuki et al., 2015). Others have demonstrated that in the aging human aorta, the degradation of the extracellular matrix is exacerbated by increased levels and activity of the inflammatory enzyme, MMP-2 (McNulty et al., 2005; Tayebjee et al., 2005). Intriguingly, in this study we observed a paradoxical decrease in aortic stiffness in the 12 month compared to 7 month mice. Counter to the previous report which demonstrated that male C57BL/6 mice thoracic aortas progressively increase in stiffness at 12 month compared to 4 month (De Moudt et al., 2022). We hypothesize that our observations could be due to a retention of elastic properties as we did not observe an age dependent change in luminal diameter as these have been described as the most consistent well-reported changes resulting in aortic stiffness (Lee and Oh, 2010). Further investigation into the underlying mechanisms of this observation is warranted but falls outside the scope of this study.

While we observed no effect of oxLDL aortic stiffness in 12 month male or female, we did observe an increase in stiffness in 7 month male thoracic aortic rings following oxLDL (Figure 7). Moreover, we observed a sex-dependent response of LOX-1 inhibition which resulted in increased 7 month male stiffness but decreased age-matched female aortic stiffness in the presence or absence of oxLDL in an endothelial dependent manner. Together these data suggest that endothelial LOX-1 may play a differential role in the maintenance of 7 month male and female thoracic aortic stiffness but diminishes with age, in our case middle age. We could hypothesize that this could be a function of pathophysiologic aging-mediated upregulation of CD36 expression as observed in this study but warrants further elucidation. It has been reported that CD36 plays a critical role in oxLDL accumulation and internalization in macrophages (Nagy et al., 1998; Yang et al., 2018) from which we hypothesize that the upregulation of CD36 with age could also in part be responsible for the age dependent effects of oxLDL/LOX-1-mediated increases inward remodeling observed in the 12 month male and females, but not 7 month mice. Moreover, due to the previously noted LOX-1-mediated vascular remodeling (Hofmann et al., 2017), we theorize that CD36 is competing for oxLDL binding resulting in increased efficacy of LOX-1 inhibition to attenuate oxLDL/LOX-1-mediated inward remodeling in 12 month mice compared to 7 month mice. Together suggesting a differential role of LOX-1 in the progression of oxLDL-mediated thoracic aortic pathology in terms of stiffness and remodeling within males and female mice across age.

We acknowledge that the complex cascade of oxLDL/LOX-1 within the thoracic aorta cannot be exactly modeled in an *ex vivo* setting. Moreover, we recognize that isolated aortic ring segments do not fully replicate the complex architecture of an intact vessel, particularly the integrated endothelial and smooth muscle layers, nor do they capture the pulsatile dynamics observed *in vivo*. However, *ex vivo* studies enable the pharmacologic evaluation of vascular reactivity. Although this method may only offer an indirect assessment of specific cellular and molecular mechanisms under oxLDL exposure, the findings can still provide insights that reflect potential observations in pathological conditions like hyperlipidemia and dyslipidemia *in vivo*.

## 5 Perspectives and significance

In conclusion, our *ex vivo* study has significantly contributed to the further elucidation of the intricate relationship between the oxLDL/LOX-1 axis and mouse thoracic aortic physiology, particularly within the contexts of biological sex and the more mature aging vasculature. We have successfully demonstrated the impact of acute *ex vivo* oxLDL exposure on distinct alterations in aortic vasoreactivity, stiffness, and remodeling, revealing differential responses across age and sexes. Importantly, our findings highlight the pivotal role of the endothelium and LOX-1 in coordinating these responses. Our results also further point towards the interplay between oxLDL and endothelin-1, contextualizing potential contributions to observed vasoreactivity changes. Furthermore, our paradoxical observation of decreased aortic stiffness in the older cohort of mice (12 month old) studied warrants deeper exploration to uncover the underlying mechanisms of oxLDL and its role in regulating vascular reactivity during middle age. While we observed varying effects of LOX-1 inhibition on stiffness between age-matched male and female mice, the LOX-1 receptors potential relationship with CD36-mediated processes adds yet another layer of complexity to the multifaceted nature of oxLDL-mediated effects. Future studies will be aimed at further elucidation into the mechanisms linking oxLDL, LOX-1, endothelin-1, and CD36, while also considering the broader implications for age and sex differences in vascular health. This study lays a strong foundation for unraveling the elaborate molecular pathways that contribute to oxLDL/LOX-1-mediated alterations in aortic physiology, with potential implications for therapeutic interventions in cardiovascular health.

## Data Availability

The original contributions presented in the study are included in the article/Supplementary Material, further inquiries can be directed to the corresponding author.
