# Supplementary material for: OxLDL/LOX-1 mediated sex, age, stiffness, and endothelial dependent alterations in mouse thoracic aortic vascular reactivity
Source: Front Physiol. 2024 Nov 5;15:1471272. doi: 10.3389/fphys.2024.1471272 (PMC11573510; doi:10.3389/fphys.2024.1471272)
Supplement: Supplementary file 1 [file DataSheet1.pdf]

frontiers

Supplementary Figures 1-5

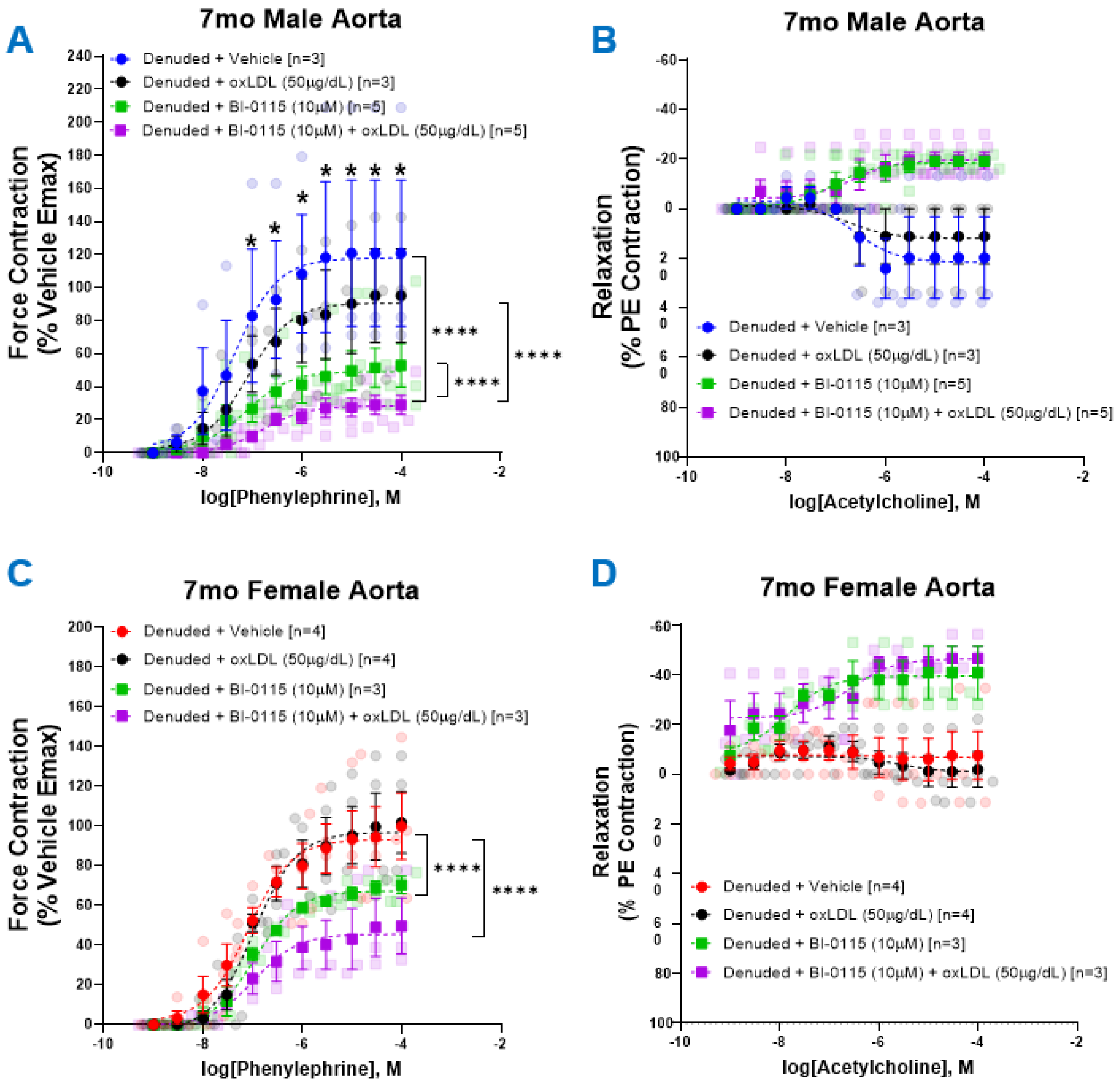

Supplemental Figure 1. Vasoreactivity of Male and Female 7-month-old Endothelial Denuded Thoracic Aortic Rings. Concentration response curves to (**A-C**) phenylephrine (PE) and (**B-D**) ACh (PE-precontracted) in 1mm denuded thoracic aortic rings following exposure to either vehicle (Male: n=3; Female: n=4), oxLDL (50μg/dL) (Male: n=3; Female: n=4), BI-0115 (10μM; selective LOX-1 inhibitor) (Male: n=5; Female: n=3), or oxLDL (50μg/dL) + BI-0115 (10μM) (Male: n=5; Female: n=3) for 2h. Individual values are shown are transparent data points. Grouped data are represented as means ± SEM. Two-Way ANOVA with Tukey's post-hoc test. *p<0.05, ****p<0.0001.

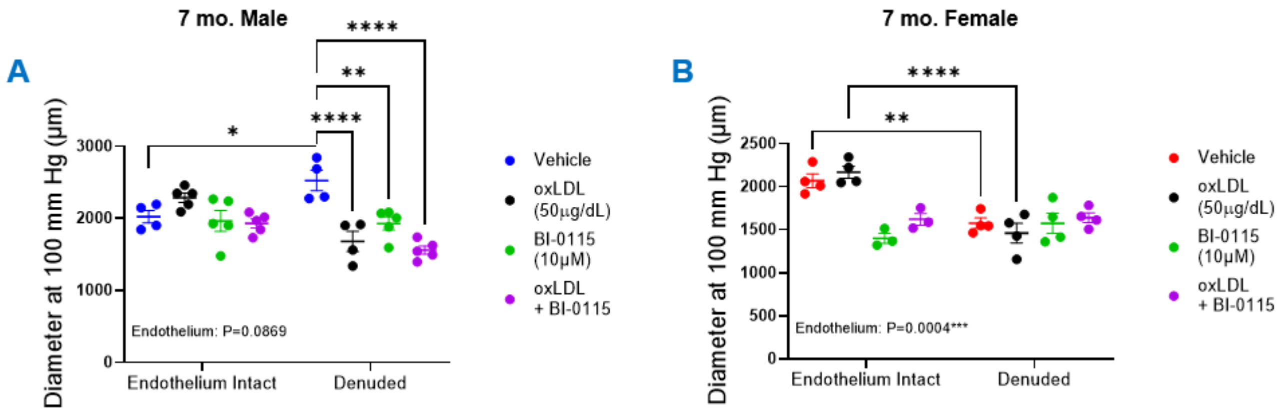

Supplemental Figure 2. Aortic Remodeling of Male and Female 7-month-old Endothelial Intact and Denuded Thoracic Aortic Rings in the Presence or Absence of LOX-1 Inhibition. Graphs depicting wire myography mediated estimated diameter at 100 mm Hg from 1mm thoracic aortic rings obtained from 7 mo. endothelial intact and denuded male (**A**) and female (**B**) mice following exposure to either vehicle (Male: n=4-5; Female: n=4-5), oxLDL (50μg/dL) (Male: n=4-5; Female: n=4-5), BI-0115 (10μM; selective LOX-1 inhibitor) (Male: n=5; Female: n=3-4), or oxLDL (50μg/dL) + BI-0115 (10μM) (Male: n=5; Female: n=3-4) for 2h. Data are represented as means ± SEM. Two-Way ANOVA with Tukey's post-hoc test. *$p<0.05$, **$p<0.01$, ***$p<0.001$, ****$p<0.0001$.

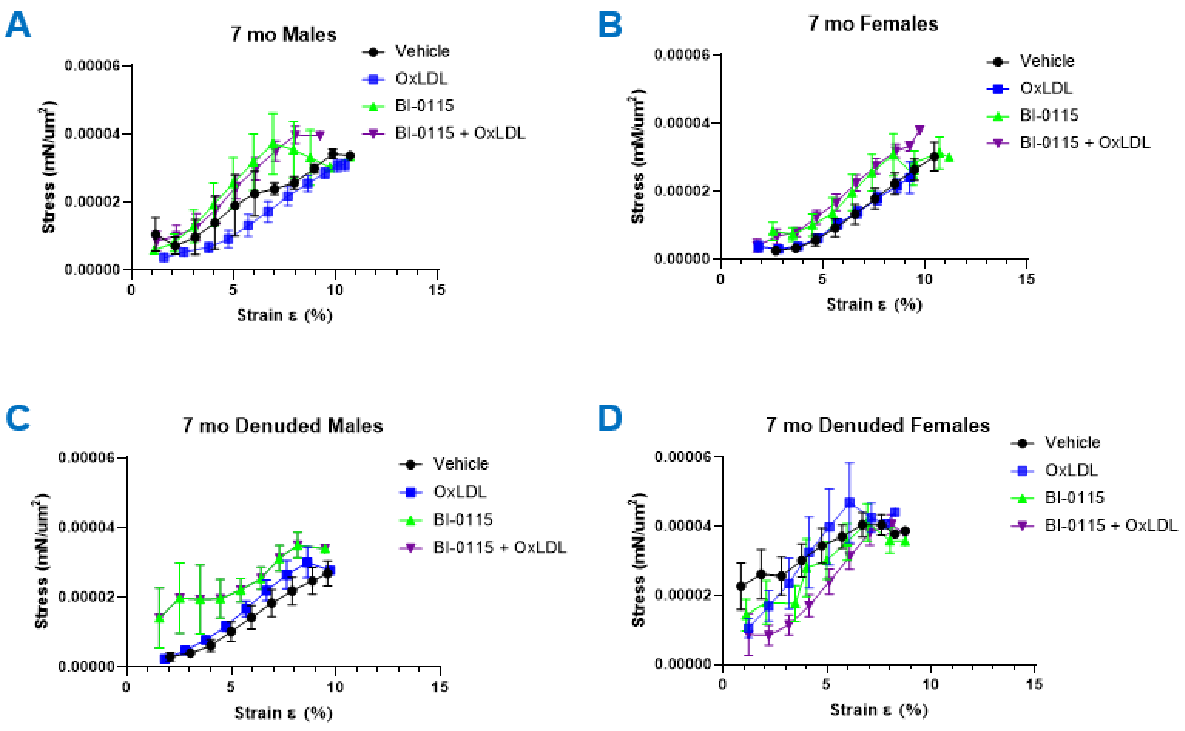

Supplemental Figure 3. Stress Strain Curves of Endothelium Intact and Denuded Thoracic Aortic Ring Segments. Aortic segments were isolated from (**A**) 7 mo. old males, (**B**) 7 mo. old females, (**C**) 12 mo. old males and D) 12 mo. females were treated for 2h ex vivo with vehicle (Veh; >0.1% DMSO), oxLDL (50µg/dL); BI-0115 (10µM; selective LOX-1 inhibitor), or oxLDL plus BI-0115. Vertical bars denote standard error of the mean. Stress is expressed at force (mN)/area (mm^2). Stain is expressed as the ratio of (initial diameter – final diameter)/final diameter.

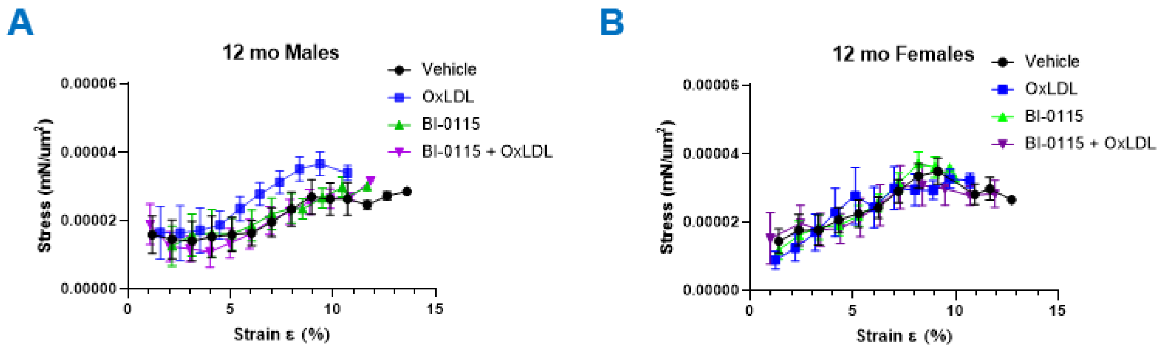

Supplemental Figure 4. Stress Strain Curves of Denuded Thoracic Aortic Ring Segments. Aortic segments isolated from (**A**) endothelium intact 12 mo. old males, (**B**) endothelium intact 12 mo. old females treated for 2h ex vivo with vehicle (Veh; >0.1% DMSO), oxLDL (50µg/dL); BI-0115 (10µM; selective LOX-1 inhibitor), or oxLDL plus BI-0115. Vertical bars denote standard error of the mean. Stress is expressed at force (mN)/area (mm^2). Stain is expressed as the ratio of (initial diameter – final diameter)/final diameter.

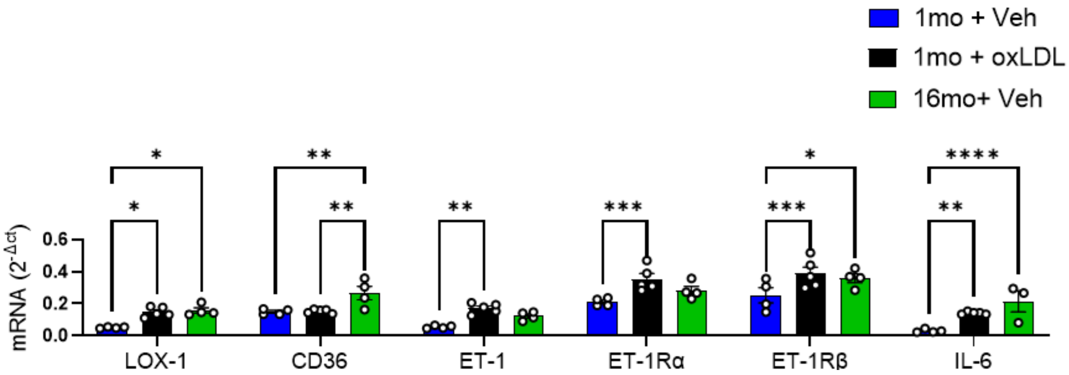

Supplemental Figure 5. OxLDL Differentially Alters Mediator mRNA Expression of Thoracic Aortic Stiffness, Remodeling, and Vasoreactivity and Pro-Inflammation. qRT-PCR graph of thoracic aortas from 1 mo. male exposed to either vehicle (n=4) or oxLDL (50µg/dL; n=5) and 16 mo. male mice exposed to vehicle (n=3-4) for 2h. Data are represented as means ± SEM. Two-Way ANOVA with Tukey's post-hoc test. *p<0.05, **p<0.01, ***p<0.001, ****p<0.0001.
